# APOEFormer: A Multimodal AI Model for APOE4 Carrier Status Prediction and Phenotype Characterization in Alzheimer’s Disease

**DOI:** 10.3390/bioengineering13101113

**Published:** 2026-09-24

**Authors:** Thong Nguyen, Carter Woods, Jian Liu, Chris Wang, Ai-Ling Lin, Jianlin Cheng

**Affiliations:** 1 Department of Electrical Engineering and Computer Science, University of Missouri, Columbia, MO 65201, USA; tmnthc@missouri.edu (T.N.); jl4mc@missouri.edu (J.L.); cwcn6@missouri.edu (C.W.); 2 Roy Blunt NextGen Precision Health, University of Missouri, Columbia, MO 65201, USA; cdw5fz@missouri.edu

**Keywords:** Alzheimer’s disease, multimodal AI, transformer, contrastive learning, self-supervised learning, data integration

## Abstract

The apolipoprotein E ε4 (APOE4) allele is the strongest genetic risk factor for late-onset Alzheimer’s disease (AD), the most common form of dementia. APOE4 carriers exhibit cerebrovascular and metabolic dysfunction, structural brain alterations, and gut microbiome changes decades before the onset of clinical symptoms. Better understanding of the early manifestation of these physiological changes is critical for development of timely AD interventions and risk reduction protocols. Multi-modal datasets encompassing a wide range of APOE ε4 and AD associated biomarkers provide a valuable opportunity to gain insight into the APOE4 phenotype; however, these datasets often present analytical challenges due to small sample sizes and high heterogeneity. Here, we propose a two-stage multimodal AI model (APOEFormer) that integrates blood metabolites, brain vascular and structural MRI, microbiome profiles, and other clinical and demographic data to predict APOE4 allele status. In the first stage, modality-specific encoders are used to generate initial representations of input data modalities, which are aligned in a shared latent space via self-supervised contrastive learning during pretraining. The contrastive learning objective encourages learning of informative and consistent representations across modalities through leveraging cross-modality relationships. In the second stage, the pretrained representations are used as inputs to a multimodal transformer that integrates information across modalities to predict a key AD-risk genetic variant (APOE4). Across 10 repeated experimental runs with different train–validation–test splits, APOEFormer predicts whether an individual carries an APOE4 allele with an average prediction accuracy of 75%, demonstrating robust performance under limited sample sizes. Post hoc perturbation analysis of the predictive model revealed valuable insights into the driving components of the APOE4 phenotype—including key blood biomarkers and brain regions strongly associated with APOE4.

## 1. Introduction

The apolipoprotein E ε4 (APOE4) allele is the strongest genetic risk factor associated with Alzheimer’s disease (AD). A single APOE4 allele increases an individual’s risk late-onset AD development by 3–7 times in heterozygous carriers while homozygous APOE4 carriers experience up to a 12-fold increase in risk of AD development [1,2]. Beyond higher risk to AD development the APOE4 allele is associated with a complex cascade of physiological effects, including metabolic dysfunction, structural brain alterations, and gut microbiome changes that occur decades before and are believed to contribute to AD development. Understanding how these diverse biomarkers coalesce in the APOE4 phenotype is essential to identify early disease mechanisms and develop precision interventions.

Deconvolution of the complex APOE4 phenotype requires a sophisticated integration of multi-modal datasets, starting with the foundational role of magnetic resonance imaging (MRI) in capturing early neuroanatomical changes. In APOE4 carriers, MRI-derived measures frequently reveal accelerated cortical thinning and cerebral blood flow, both measures connected to neurodegeneration [3,4]. Complementary non-imaging biomarkers—including biochemical measurements, microstructural indicators, and clinical variables—encode additional aspects of disease pathology that are not directly observable in imaging data [5,6]. Moreover, recent research revealed that the bidirectional interaction between gut microbiome and brain plays an important role in AD [7].

Recent studies further support the presence of heterogeneous phenotypic alterations associated with APOE4. Mares et al. reported APOE4-associated heterogeneity across multiple neuroimaging biomarkers, including amyloid deposition, tau accumulation, glucose metabolism, brain volume, cerebral blood flow, and white-matter hyperintensities [8]. A recent systematic review also identified measurable APOE4-associated neuroimaging differences in cognitively healthy adults during midlife [9]. In addition, recent metabolomic analyses have identified APOE4-associated metabolic alterations in plasma and brain tissue [10]. Together, these findings further motivate the characterization of APOE4 as a heterogeneous multimodal phenotype rather than through any single imaging or molecular marker.

Traditional machine learning approaches for multimodal phenotype analysis typically rely on handcrafted feature extraction followed by shallow fusion strategies, such as feature concatenation or ensemble classifiers [11]. While these methods have shown some success, they often struggle with high-dimensional inputs, limited sample sizes, and complex nonlinear interactions between modalities. Moreover, differences in scale, noise characteristics, and missing values across modalities can lead to poorly aligned representations and reduced generalization performance.

Deep learning methods have recently emerged as powerful alternatives for multimodal biomedical data integration. Convolutional neural networks have been widely applied to MRI-based AD classification [12], while multilayer perceptrons (MLPs) and autoencoder-based architectures have been used to model tabular clinical and biomarker data [13]. Despite these advances, many existing deep multimodal models are trained end-to-end using supervised objectives alone, making them vulnerable to overfitting in settings where labeled data is scarce or heterogeneous across cohorts.

Self-supervised and contrastive learning frameworks provide a principled approach to addressing these limitations by enabling representation learning from unlabeled or weakly labeled data. Contrastive objectives encourage embeddings of different views or modalities from the same subject to be similar, while maintaining discriminative separation across subjects [14,15]. Such approaches have demonstrated strong performance in vision, language, and biomedical domains [16], particularly when annotations are limited or expensive to obtain. In multimodal medical settings, contrastive learning has been shown to effectively align imaging and non-imaging representations into a shared latent space, improving downstream predictive performance and robustness [17].

In parallel, transformer-based architectures have become increasingly influential due to their ability to model complex dependencies using attention mechanisms [18]. Transformers allow models to dynamically weight informative features and capture long-range interactions without relying on fixed receptive fields [18]. These properties are particularly advantageous for biomedical data, where disease-relevant signals may be distributed across spatially distant brain regions or across heterogeneous biomarker sets. Recent studies have successfully applied transformer-based models to medical imaging, clinical time series, and multimodal health data, demonstrating improved flexibility and interpretability compared to conventional architectures [19,20].

Recent work has further demonstrated the potential of transformer-based and contrastive-learning strategies for multimodal AD analysis. Yu et al. developed a unified transformer-based framework integrating structural MRI with clinical and genetic information for AD assessment [21]. More recently, multimodal contrastive learning has been applied to align heterogeneous representations and improve AD classification [22]. These developments highlight the growing importance of representation alignment and attention-based fusion for integrating heterogeneous biomedical modalities.

In this work, we introduce a two-stage multimodal AI framework (APOEFormer) for APOE4 carrier prediction and phenotype characterization that combines contrastive representation learning with attention-based multimodal integration. In the first stage, modality-specific encoders are used to generate the representations of data modalities, which are aligned in a unified latent space by contrastive learning during self-supervised pretraining. This stage enables the model to learn robust, aligned representations of the heterogeneous modalities that capture phenotypical features without relying on labeled data. In the second stage, the pretrained representations are used as inputs to a transformer-based model that integrates information across modalities to predict whether a subject carries at least one APOE4 allele. Because the input representations are of high quality, fine-tuning the transformer model only needs a small number of labeled data samples.

The methodological novelty of APOEFormer lies in its two-stage strategy for integrating highly heterogeneous biomedical modalities in a limited-sample setting. Modality-specific representations are first aligned through contrastive pretraining, an approach that has been increasingly used to learn shared representations across heterogeneous biomedical modalities [15,17], and are subsequently integrated using transformer-based self-attention [18], rather than relying solely on direct feature concatenation. The framework is further evaluated through conventional machine-learning baselines and a contrastive-pretraining ablation to assess whether the proposed architecture provides benefits beyond simpler alternatives. Furthermore, APOEFormer combines prediction with modality-, brain-region-, and biomarker-level interpretation, enabling characterization of the multimodal phenotypic patterns associated with APOE4.

By decoupling the representation learning from downstream predictive modeling, the proposed framework addresses key challenges in multimodal APOE4 phenotype analysis, including data heterogeneity, label scarcity, and complex cross-modal interactions.

Moreover, the prediction of the AI model is further interpreted by the post hoc perturbation technique to identify key modalities and features associated with APOE4 variant, providing valuable insights about AD vulnerability. Therefore, APOEFormer provides a scalable and extensible approach to multimodal learning for APOE4 carrier-status prediction and characterization that can generalize to a broader class of disease-associated biomarker classification tasks.

## 2. Materials and Methods

### 2.1. APOEFormer Overview

APOEFormer is a multimodal AI framework designed to integrate heterogeneous subject data, such as neuroimaging and clinical biomarkers, into a unified representation for predicting an individual’s APOE4 carrier status.

The architecture of APOEFormer (Figure 1) follows a two-stage learning paradigm that decouples representation learning from downstream prediction tasks. In the first stage, modality-specific encoders are trained using a contrastive learning objective to project imaging and non-imaging data modalities into a shared latent space. Structural MRI volumes and perfusion images are encoded using a CLIP-based vision encoder [16], leveraging pretrained visual representations that have demonstrated strong transferability to medical imaging tasks [16,17]. In parallel, heterogeneous non-imaging modalities—including microstructural features, multiple biomarker panels, and numeric clinical variables—are encoded using multilayer perceptron (MLP)-based networks. This design allows each modality to preserve domain-specific inductive biases while producing metrically aligned embeddings through self-supervised contrastive learning [14,15].

The self-supervised contrastive pretraining is particularly well suited to phenotype modeling because it does not require labels, where labeled outcomes are often limited, heterogeneous across cohorts, or affected by diagnostic uncertainty [23]. By aligning multimodal embeddings within the same subject and across subjects, the model learns improved representations of heterogeneous data modalities by leveraging modality relationships, while remaining robust to noise. The resulting embeddings serve as standardized representations for downstream modeling, analogous to representation alignment strategies used in self-supervised language and vision transformers [16,24].

In the second stage, APOEFormer employs a transformer-based fusion module to integrate pretrained embeddings across modalities. Attention mechanisms enable the model to dynamically capture interactions among imaging and non-imaging features, allowing it to adaptively weight informative signals across modalities [18]. This flexibility is especially important in phenotype modeling, where the relative contributions of structural brain changes and non-imaging biomarkers may vary across individuals and disease stages.

The transformer encoder produces contextualized subject-level representations that summarize multimodal disease information. These representations are aggregated through attention-based pooling and passed to a prediction head that outputs a binary classification indicating whether a given patient carries the APOE4 allele. APOE4 is the strongest known genetic risk factor for late-onset AD and is associated with accelerated neurodegeneration, altered biomarker profiles, and increased disease susceptibility [25]. Predicting APOE4 carrier status from multimodal phenotypic data provides a biologically grounded and clinically meaningful target that links genetic risk to observable neuroimaging and clinical patterns.

By integrating contrastive self-supervised learning with transformer-based multimodal modeling, APOEFormer addresses key challenges in phenotype analysis, including data heterogeneity, limited labels, and complex cross-modal interactions. The proposed framework provides a scalable and extensible foundation for studying genetic risk variants and their effects in AD and can be generalized to study other diseases with multimodal data.

The components of APOEFormer, learning strategies, and methods to interpret predictions are described in the subsections below.

### 2.2. Image Encoder for Encoding MRI Images

Structural MRI and Perfusion image volumes provide rich anatomical information but pose challenges for representation learning due to their high dimensionality and variability in slice count across subjects. Direct 3D convolutional modeling is often data-intensive and sensitive to limited cohort sizes [26]. To address these challenges, we adopt a slice-wise encoding strategy that leverages large-scale pretrained visual representations generated by CLIP ViT-B/32 [16] while preserving depth-wise anatomical structure. Each MRI and Perfusion volume is decomposed along the axial dimension into an ordered sequence of 2D slices, enabling the use of powerful 2D vision transformers without discarding volumetric context.

Each slice undergoes intensity normalization to mitigate scanner-dependent variability and numerical instability arising from low-contrast or near-zero-intensity regions. The normalized slices are converted into three-channel images and optionally augmented during training to improve robustness. Feature extraction is performed using the vision encoder of CLIP ViT-B/32, initialized from large-scale image–text pretraining [16]. Given the limited size typical of biomedical datasets, most CLIP parameters are frozen to stabilize optimization, while only the final transformer blocks are unfrozen to allow controlled task-specific adaptation. A gradual unfreezing strategy is employed, enabling additional layers to be released when validation performance plateaus, thereby balancing representation flexibility and generalization.

The high-dimensional CLIP embeddings are then projected into a compact latent space using a lightweight projection head composed of linear transformations, normalization, and nonlinear activation. Both the CLIP features and projected embeddings are $\ell_{2}$-normalized to enforce consistent scale across slices and modalities. The slice encoding process can be summarized as

(1) $$z_{i,d}=\mathrm{Proj}\left(\mathrm{CLIP}(x_{i,d})\right),$$

where $x_{i,d}$ denotes the *d*-th slice of subject *i* and $z_{i,d}$ is the corresponding slice embedding. The resulting embeddings form a depth-ordered sequence that is padded or truncated to enable batch-wise processing while preserving relative slice structure.

### 2.3. MLP Encoder for Non-Image Modalities

In addition to imaging data, our framework incorporates multiple non-imaging modalities, including gut microbiome profiles, CBF value, blood-based biomarker panels, auxiliary clinical variables (e.g., gender, age, and body mass index [BMI]). These modalities encode complementary biological and phenotypic information but differ substantially in dimensionality, statistical distribution, and semantic meaning. Direct concatenation of such heterogeneous inputs can lead to scale imbalance and suboptimal feature interactions, particularly in attention-based fusion architectures [11].

To address this challenge, each non-imaging modality is processed using a dedicated multilayer perceptron (MLP) encoder that maps raw inputs into a latent embedding space. Each encoder consists of stacked transformation blocks comprising a linear projection, layer normalization, nonlinear activation, and dropout. Layer normalization is used to reduce covariate shift and improve training stability across modalities, while dropout provides regularization against overfitting [27]. Residual connections are applied whenever dimensionality permits, allowing the encoder to preserve informative low-level signals while learning higher-level abstractions.

A final normalization step followed by $\ell_{2}$-normalization ensures that all modality embeddings lie on a comparable scale, facilitating stable cross-modal interaction during attention-based fusion. For a given modality input vector $x_{i}$, the encoding process can be written as

(2) $$e_{i}=\mathrm{MLP}\left(x_{i}\right),$$

where $e_{i}$ denotes the normalized modality embedding. By enforcing a consistent embedding dimensionality and normalization scheme across modalities, these MLP encoders provide a principled interface for integrating heterogeneous biomedical data.

### 2.4. Contrastive Learning for Modality Alignment During Pretraining

Learning robust representations from heterogeneous biomedical data is challenging due to substantial variation in scale, noise characteristics, and semantic meaning across modalities. In multimodal clinical settings, imaging-derived features, molecular profiles, and structured clinical variables provide complementary but weakly aligned views of the same subject. To encourage coherent cross-modal representations prior to transformer fusion, we employ a contrastive learning-based pretraining strategy that explicitly aligns modality-specific embeddings at the subject level (Figure 1). During pretraining, each subject is represented by multiple modality embeddings, including imaging-based embeddings and non-imaging embeddings produced by modality-specific MLP encoders. All embeddings are normalized to lie in a shared metric space, enabling direct similarity comparisons across modalities. The contrastive objective follows an InfoNCE-style formulation, in which subject identity provides supervision: embeddings derived from different modalities of the same subject are treated as positive pairs, while embeddings originating from different subjects are treated as negatives [14], regardless of modality. This results in a multi-positive contrastive setting, where each anchor embedding is contrasted against all other modality embeddings within the minibatch.

Pairwise similarities are computed using temperature-scaled cosine similarity, and the InfoNCE normalization encourages embeddings associated with the same subject to form compact clusters while maintaining separation between subjects in the latent space. By aligning heterogeneous modalities through this objective, the model learns modality-invariant representations that capture subject-specific characteristics rather than modality-specific artifacts.

Formally, the contrastive objective is defined as

(3) $$L_{\mathrm{ctr}}=E_{i}\left[-\log\frac{\sum_{j\in P(i)}\exp\left(\mathrm{sim}\left(z_{i},z_{j}\right)/\tau \right)}{\sum_{k\neq i}\exp\left(\mathrm{sim}\left(z_{i},z_{k}\right)/\tau \right)}\right],$$

where $z_{i}$ denotes a modality-specific embedding, P(i) is the set of embeddings corresponding to the same subject (i.e., positive pairs), sim(·,·) denotes cosine similarity, and τ is a temperature hyperparameter controlling the sharpness of the similarity distribution. The expectation $E_{i}$ denotes averaging over all anchor embeddings *i* within a mini-batch.

The objective $L_{\mathrm{ctr}}$ is minimized during training. Minimizing this loss increases similarity between embeddings belonging to the same subject (maximizing the numerator) while reducing similarity to embeddings from different subjects (implicitly minimizing the denominator’s relative contribution), thereby promoting intra-subject compactness and inter-subject separation in the latent space.

This contrastive pretraining phase produces a shared embedding space in which representations from different modalities are aligned at the subject level, providing a robust initialization for subsequent transformer-based multimodal fusion and downstream prediction.

### 2.5. Multimodal Transformer for APOE4 Carrier-Status Prediction

To integrate the aligned representations of multiple modalities into a unified subject-level representation to predict APOE4 carrier status, we employ a transformer built on stacked self-attention layers [18] (Figure 1). All modality embeddings, including MRI and Perfusion images slice embeddings and static tabular embeddings, are treated as tokens within a single sequence. This formulation allows the model to learn context-dependent interactions across slices and modalities, capturing dependencies that are difficult to model using fixed aggregation strategies.

Each self-attention layer applies multi-head attention followed by dropout, residual connections, and layer normalization. This architecture enables stable gradient flow and progressively refines token representations by allowing each embedding to attend to all others. By stacking multiple attention layers, the transformer can capture higher-order cross-modal interactions and dynamically reweight modalities based on their relevance to the prediction task.

Following attention-based contextualization, the variable-length token sequence is compressed into a fixed-dimensional subject representation using a learned attention pooling mechanism [28]. Each token is assigned an importance score, and the final representation is computed as a weighted sum of token embeddings:

(4) $$r_{i}=\sum_{t}\alpha_{i,t}s_{i,t},$$

where $s_{i,t}$ denotes the contextualized embedding of token *t* for subject *i*, and $\alpha_{i,t}$ are learned attention weights. The pooled representation is subsequently passed through a lightweight MLP to produce the final prediction. This combination of self-attention and learned pooling enables the model to emphasize relevant slices or modalities while suppressing less informative inputs.

### 2.6. Supervised Transformer Training

Following the contrastive multimodal pretraining, the model is trained in a supervised manner to predict APOE4 carrier status at the subject level. In this phase, the pretrained modality encoders generate fixed-dimensional embeddings for each subject, which are then provided as input tokens to the transformer classifier. The transformer serves as the primary decision module, learning to integrate information across modalities and to produce a discriminative subject-level representation tailored to APOE ε4 prediction.

Supervised training is conducted using labeled subjects, with strict separation of training, validation, and test sets to prevent information leakage across individuals. For each subject, multimodal embeddings are assembled into a token sequence and processed by a stack of self-attention layers. Residual connections, dropout, and layer normalization are applied throughout the transformer, enabling stable optimization and allowing the model to dynamically reweight modalities based on their relevance to APOE4 status.

To address class imbalance and heterogeneous prediction difficulty inherent to APOE4 classification, the transformer is optimized using a focal loss objective [29]. Focal loss extends binary cross-entropy (BCE) by introducing multiplicative weighting factors that reduce the contribution of well-classified samples while emphasizing harder examples. This property is particularly important in clinical genetics settings where carrier prevalence is uneven.

Specifically, the supervised training objective is defined as

(5) $$L_{\sup}=\alpha_{t}{\left(1-p_{t}\right)}^{\gamma }\;\mathrm{BCE}\left(y,\hat{y}\right),$$

where y∈{0,1} denotes APOE4 carrier status, $\hat{y}$ denotes the predicted probability of the positive class, and

$$p_{t}=\left\{\begin{array}{cc} \hat{y}, & y=1,\\ 1-\hat{y}, & y=0. \end{array}\right.$$

The binary cross-entropy term is given by

(6) $$\mathrm{BCE}\left(y,\hat{y}\right)=-\left[y\log\left(\hat{y}\right)+\left(1-y\right)\log\left(1-\hat{y}\right)\right].$$

Here, the three components in Equation (5) are multiplicative rather than additive: $\mathrm{BCE}(y,\hat{y})$ provides the base binary classification loss, $\alpha_{t}$ is a class-balancing coefficient that adjusts the relative weight of carriers and non-carriers, and the factor ${\left(1-p_{t}\right)}^{\gamma }$ modulates the loss such that confidently classified samples contribute less to the overall objective. The focusing parameter γ controls the strength of this modulation.

Training is performed using the AdamW optimizer with weight decay, gradient clipping, and mixed-precision arithmetic to ensure numerical stability and computational efficiency. Early stopping based on validation loss is employed to mitigate overfitting, and the best-performing transformer model is selected for final evaluation.

The main architectural and training hyperparameters used in APOEFormer are summarized in Table 1.

### 2.7. Multimodal Input Data

The multimodal data analyzed in this study were obtained from the EXPRESS pilot trial, which investigated low-dose rapamycin in cognitively normal middle-aged adults stratified by APOE genotype, as described in detail in the original publication [30]. The study cohort consisted of 23 participants, including 9 APOE4 carriers and 14 non-carriers. The dataset spans neuroimaging, molecular, microbial, and clinical domains, enabling characterization of both systemic and brain-specific biological processes. Structural MRI features were derived from high-resolution T1-weighted images, which provide detailed anatomical characterization of cortical thickness, subcortical volumes, and gray–white matter boundaries. Structural MRI has been widely used for quantifying neurodegeneration and regional atrophy patterns in aging and neurological disorders [31]. In addition to structural anatomy, functional perfusion information was incorporated through perfusion image, obtained using arterial spin labeling (ASL). ASL is a non-invasive MRI technique that quantifies cerebral blood flow (CBF) by magnetically labeling inflowing arterial blood water, enabling voxel-wise perfusion mapping without contrast agents [32].

To clearly distinguish image-based perfusion representations from derived quantitative summaries, we further included CBF value, defined as tabular region-averaged CBF measurements extracted from anatomically parcellated perfusion maps. This separation allows the model to independently leverage high-dimensional spatial perfusion patterns and lower-dimensional region-level perfusion metrics.

Beyond neuroimaging, multiple blood-derived modalities were incorporated. Blood metabolites consisted of quantified plasma metabolomic profiles, capturing downstream biochemical activity reflective of systemic metabolic states. Inflammatory markers denote circulating inflammatory biomarker levels measured from plasma, providing insight into immune and inflammatory processes that may influence neurological and systemic health. CBC value included routine clinical laboratory measurements, offering standardized indicators of organ function and physiological status. The Microbiome modality comprised gut microbial taxonomic abundances, representing host–microbe interactions that have increasingly been linked to neuroinflammation and brain function through the gut–brain axis. Finally, the Demographic/clinical data category included demographic and auxiliary structured variables such as age, sex, and additional clinical covariates.

Collectively, this multimodal design enables the model to integrate complementary structural MRI, perfusion-based, metabolic, inflammatory, microbial, and demographic information, thereby capturing both localized neurobiological alterations and systemic physiological states. Such integrative approaches are increasingly recognized as essential for modeling complex brain–body interactions in translational biomedical research.

All modalities included in the present analysis were available for all 23 subjects; therefore, there were no missing modality-level data within the analyzed cohort.

### 2.8. SHAP Analysis for Modality Importance

To quantify the contribution of each modality within the multimodal integration model, we applied SHAP (SHapley Additive exPlanations), a feature-attribution framework based on Shapley values from cooperative game theory [33]. Specifically, we used KernelSHAP implemented through the SHAP KernelExplainer (version 0.52.0), which provides a model-agnostic approximation of Shapley values by evaluating different combinations of input features.

The learned modality embeddings were used as inputs to the SHAP analysis, with an all-zero embedding vector serving as the background/reference input. For each patient, SHAP values were estimated using 200 perturbation samples. The absolute SHAP values were then averaged across patients and embedding dimensions to obtain a single importance score for each modality.

The resulting mean absolute SHAP value represents the relative contribution of each modality to the model’s prediction in the multimodal integration setting. A larger mean absolute SHAP value indicates that the corresponding modality has a greater influence on the model output.

### 2.9. Model Interpretation: MRI Brain Region Importance Analysis

To investigate the neuroanatomical patterns underlying APOEFormer’s APOE4 predictions, we conducted a brain region importance analysis based exclusively on structural MRI imaging data. This analysis was performed only on MRI-derived features, and did not involve perfusion imaging modalities. The goal was to identify MRI brain regions whose structural perturbation most strongly influences the model’s predictions, while controlling for confounding effects introduced by regional size differences.

For each MRI-derived brain parcel, regional importance was estimated using a masking-based perturbation strategy. Specifically, for a given parcel, a fixed-size subregion was repeatedly masked, and the resulting change in the model’s prediction score was measured. This procedure was repeated 100 times per parcel. Within each parcel, the location of the fixed-size mask was randomly sampled across the 100 perturbation repetitions. Therefore, the resulting regional importance score reflects the average sensitivity of the model to localized perturbations distributed throughout the parcel, rather than the effect of masking a single fixed sub-region. The average change in prediction score across the 100 repetitions was then used as the parcel’s importance estimate. By using a constant mask size across all regions, this approach isolates the effect of localized structural perturbations rather than differences in overall parcel volume.

The smallest parcel in the MRI parcellation (“right vessel”) contained very few voxels and produced negligible changes in prediction score, rendering it barely visible in visualization tools such as Mango (version 4.1). Instead, the second-smallest parcel (“right lateral ventricle”) was used to define the reference mask size, ensuring stable and consistent normalization across all MRI regions.

Prior to normalization, white matter regions dominated the importance ranking, as larger and more spatially extensive parcels naturally induced larger prediction changes when masked. This behavior reflects a known limitation of unnormalized perturbation analyses, in which region size strongly biases importance estimates. After applying size-normalized masking, however, the importance distribution shifted toward functionally and clinically relevant cortical regions, revealing a more interpretable pattern of MRI-based contributions.

### 2.10. Model Interpretation: Non-Imaging Feature Importance Analysis

To elucidate the biochemical and physiological signals driving APOEFormer’s APOE4 predictions, we conducted a feature-level importance analysis focusing on non-imaging inputs, including blood-based CBC value profiles and standard blood metabolites measurements. This analysis aimed to identify individual non-imaging features whose perturbation most strongly influences the model’s output and to assess whether the identified signals are consistent with known biological mechanisms implicated in AD.

Feature importance for non-imaging modalities was estimated using a unified masking-based perturbation strategy applied to both metabolomic and blood laboratory data. For each feature, its value was individually set to zero in the original feature space before z-score normalization was applied, while all other input features and modalities were left unchanged. The perturbed input was then processed through the same normalization and model-prediction pipeline as the corresponding unperturbed input. Therefore, the zero value used for perturbation represents zero in the original feature space rather than the mean of the standardized feature distribution. The resulting change in the model’s prediction score for APOE4 status was measured relative to the unperturbed baseline. The mean change in prediction score was then computed for each feature, yielding a model-agnostic estimate of relative feature contribution.

Perturbation-based feature ablation has been widely used to interpret complex machine-learning models in biomedical applications because it does not rely on internal gradients or assumptions about linearity and remains applicable to heterogeneous architectures [34,35,36]. Applying an identical perturbation protocol across heterogeneous input modalities enables consistent comparison of feature sensitivity within multimodal transformer frameworks.

## 3. Results

### 3.1. The Accuracy of Predicting APOE4

To evaluate the robustness of APOEFormer under varying data partitions, we conducted 10 experimental runs using different splits of the 23 patients into training, validation, and test sets. Among the 23 patients, 9 patients (39.1%) were APOE4 carriers and 14 patients (60.9%) were non-carriers. In each run, 16 patients were used for training, 3 for validation, and 4 for testing. The training, validation, and test sets were mutually exclusive within each run, although patients could appear in different subsets across runs due to repeated re-sampling. The test set was selected using stratified sampling to maintain a balanced representation of APOE4-positive and APOE4-negative patients.

Importantly, all model training procedures, including contrastive pretraining, were performed exclusively using patients in the training set of the corresponding run. Therefore, patients assigned to the validation or test sets were not observed during either contrastive pretraining or supervised model training.

Regarding the overlap between training sets across runs, we quantified the pairwise overlap among the 10 selected experimental runs. Each run contained 16 training patients. Across the 45 possible pairs of runs, the number of shared training patients ranged from 9 to 13, with a mean overlap of 11.07 patients. This corresponds to an average overlap of approximately 69.2% of each 16-patient training set. Therefore, the repeated runs should not be interpreted as statistically independent evaluations, and the observed run-to-run variability reflects differences in patient-level data partitions within the same cohort.

Therefore, the 10 runs are not statistically independent replicates. Rather, they represent repeated evaluations using different patient-level partitions drawn from the same 23-patient cohort. Accordingly, the reported variability across runs should be interpreted as sensitivity to different train/validation/test assignments within the same cohort rather than variability across independent patient populations.

Across the 10 runs, APOEFormer achieved a mean test accuracy of 75.0%. Individual test accuracies ranged from 50% to 100% (Figure 2). Perfect classification performance was obtained in four runs (Runs 1, 4, 5, and 10), while Runs 11 and 14 achieved 75% accuracy. The remaining four runs (Runs 6, 9, 17, and 19) achieved 50% accuracy. Because each test set contained only four patients, a single incorrect prediction changed the accuracy by 25 percentage points, contributing to the observed variability across runs.

To quantify the uncertainty associated with the mean performance across the 10 repeated runs, approximate 95% confidence intervals (CIs) were calculated using the Student’s *t* distribution, which is appropriate when estimating a mean from a relatively small number of observations and the population variance is unknown [37]. For a performance metric *x*, the confidence interval was calculated as

(7) $${\mathrm{CI}}_{95\%}=\bar{x}\pm t_{0.975,\;n-1}\left(\frac{s}{\sqrt{n}}\right).$$

Because the repeated runs were generated from overlapping subsets of the same patient cohort, these observations are not fully independent. Consequently, the conventional *t*-based intervals should be interpreted as approximate descriptive measures of uncertainty across the repeated data partitions rather than confidence intervals derived from independent patient populations. Dependence among repeated training/test partitions can lead conventional variance estimates to underestimate uncertainty [38].

In addition to classification accuracy, APOEFormer was evaluated using F1 score, area under the receiver operating characteristic curve (AUROC), area under the precision–recall curve (AUPRC), sensitivity, specificity, and balanced accuracy. APOE4 carriers were defined as the positive class and non-carriers as the negative class. Sensitivity measured the proportion of APOE4 carriers correctly identified by the model, whereas specificity measured the proportion of non-carriers correctly classified. Balanced accuracy was calculated as the average of sensitivity and specificity, thereby giving equal weight to performance in both classes.

To further characterize the variability in model performance across different patient-level partitions, Table 2 summarizes the mean, standard deviation, range, and approximate 95% confidence interval for each performance metric across the 10 experimental runs.

Across the 10 runs, the mean sensitivity was 0.5500, the mean specificity was 0.9500, and the mean balanced accuracy was 0.7500. These class-specific metrics indicate that the model showed higher specificity for correctly identifying APOE4 non-carriers than sensitivity for identifying APOE4 carriers. The balanced accuracy additionally provides a class-balanced assessment of predictive performance, which is useful given the unequal overall distribution of APOE4 carriers and non-carriers in the cohort. Because each test set contained two APOE4 carriers and two non-carriers, accuracy and balanced accuracy were identical for each of the selected runs.

Across the 10 runs, APOEFormer achieved an accuracy of 0.7500 ± 0.236, an F1 score of 0.5833 ± 0.439, an AUROC of 0.7250 ± 0.343, and an AUPRC of 0.8500 ± 0.192. The relatively large standard deviations and wide confidence intervals are consistent with the small cohort size and the substantial variability across patient-level partitions.

Across the repeated experiments, the union of all test sets covered all 23 patients, so every patient was evaluated as a held-out test subject at least once. This provided broader subject-level coverage than evaluation using a single fixed test partition.

To assess the calibration of the predicted APOE4 probabilities, we additionally calculated the Brier score [39] using aggregated held-out predictions at the patient level. For patients that appeared in the test set in multiple experimental runs, their predicted APOE4 probabilities were averaged across all held-out appearances to obtain a single out-of-sample probability for each patient. Because the union of the test sets across the 10 runs included all 23 patients, this procedure yielded one aggregated held-out probability for every patient in the cohort. The Brier score was calculated as

(8) $$\mathrm{Brier}\;\mathrm{Score}=\frac{1}{N}\sum_{i=1}^{N}{\left(p_{i}-y_{i}\right)}^{2},$$

where $p_{i}$ represents the aggregated held-out predicted probability for patient *i*, $y_{i}$ represents the corresponding true APOE4 label, and N=23. The resulting Brier score was 0.1801. The Brier score ranges from 0 to 1, with lower values indicating lower probabilistic prediction error and better agreement between predicted probabilities and observed outcomes.

### 3.2. Subject-Level Statistical Evaluation

Because the 10 repeated experimental runs were generated from the same cohort, some patients appeared in the held-out test set in more than one run. To avoid treating these repeated test appearances as independent observations, we aggregated the held-out prediction probabilities at the patient level. For each patient, the predicted probabilities obtained across all of that patient’s test-set appearances were averaged, and the resulting mean probability was converted to a binary prediction using the same classification threshold of 0.5. This procedure yielded one subject-level prediction for each of the 23 patients in the cohort.

Using these aggregated predictions, APOEFormer correctly classified 18 of 23 patients, corresponding to a subject-level accuracy of 78.3%. The exact 95% binomial confidence interval for this accuracy was 56.3–92.5%. To assess whether the observed subject-level performance exceeded chance-level classification, we performed a one-sided exact binomial test against a null accuracy of 50%. The resulting *p*-value was 0.0053, indicating that the subject-level accuracy was significantly greater than the 50% chance level.

Because the cohort was imbalanced, with 9 APOE4 carriers and 14 non-carriers, we also compared APOEFormer against a majority-class baseline that classified all patients as APOE4 non-carriers. This baseline achieved an accuracy of 60.9%. An exact McNemar test was used to compare the paired patient-level predictions of APOEFormer and the majority-class classifier. APOEFormer was correct while the majority-class baseline was incorrect for five patients, whereas the majority-class baseline was correct while APOEFormer was incorrect for one patient. The corresponding two-sided exact McNemar *p*-value was 0.2188.

As summarized in Table 3, the subject-level analysis indicates that APOEFormer performs significantly better than a 50% chance-level reference. However, the improvement relative to the majority-class baseline was not statistically significant in the current cohort. Therefore, although the subject-level results provide additional evidence that the model captures predictive signal beyond random classification, the statistical findings should be interpreted cautiously given the small sample size and the imbalance between APOE4 carriers and non-carriers.

### 3.3. Comparison with Conventional Machine-Learning Baselines

To determine whether the performance of APOEFormer could be reproduced using simpler classification approaches, we compared the proposed model with three conventional machine-learning baselines: logistic regression, a multilayer perceptron (MLP), and random forest. Logistic regression provides a standard linear probabilistic model for binary classification [40], while MLPs use nonlinear hidden representations learned through backpropagation [41]. Random forest provides a nonlinear ensemble-based alternative by combining predictions from multiple decision trees [42]. All baseline models were evaluated using the same patient-level training, validation, and test partitions as APOEFormer within each of the 10 experimental runs. Therefore, within each run, all four methods were evaluated using the same held-out test patients.

For each patient, the numerical features obtained at the Baseline and Post timepoints were concatenated to form a single patient-level feature vector. Features that were constant across the training patients were removed using only the training set to prevent information leakage. For logistic regression and MLP, the remaining features were standardized using a standard scaler fitted exclusively to the training data. The fitted transformation was subsequently applied to the validation and test sets. Random forest was trained using the original feature values because tree-based models do not require feature standardization.

For logistic regression, an L2-regularized binary logistic regression classifier was trained using balanced class weights to account for class imbalance. The maximum number of optimization iterations was set to 5000. The inverse regularization parameter, *C*, was selected from 0.01,0.1,1.0,10.0 using the validation set. For each candidate value of *C*, the model was fitted only on the training patients and evaluated on the validation patients using binary log loss. The value producing the lowest validation loss was then retained for evaluation on the held-out test set.

The MLP consisted of two fully connected hidden layers containing 128 and 64 neurons, respectively, with rectified linear unit (ReLU) activation functions. The network was optimized using Adam with a learning rate of $1\times 10^{-3}$ and an L2 regularization parameter of $\alpha =1\times 10^{-4}$. Training was performed for a maximum of 1000 epochs. After each epoch, binary log loss was evaluated on the validation set. Early stopping was applied with a patience of 50 epochs and a minimum improvement threshold of $1\times 10^{-4}$, and the model parameters corresponding to the lowest validation loss were restored before test evaluation.

For random forest, an ensemble of 500 decision trees was trained using balanced class weighting. The maximum tree depth was selected from 2,3,5,None using the validation patients. Each candidate configuration was trained on the training set and evaluated using validation binary log loss. The tree depth yielding the lowest validation loss was selected for final evaluation on the corresponding test set.

For all three baseline models, the held-out test set was evaluated only after preprocessing and hyperparameter selection had been completed using the training and validation data. Predicted probabilities were converted into binary APOE4 predictions using a fixed classification threshold of 0.5, consistent with the evaluation procedure used for APOEFormer. Performance was assessed using accuracy, F1 score, area under the receiver operating characteristic curve (AUROC), and area under the precision–recall curve (AUPRC). The complete training, validation, and testing procedure was repeated across the same 10 patient-level data partitions used for APOEFormer.

Table 4 compares the predictive performance of APOEFormer with logistic regression, MLP, and random forest across the 10 patient-level data splits. APOEFormer achieved the highest mean accuracy, F1-score, AUROC, and AUPRC among the four methods, with values of 0.7500, 0.5833, 0.7250, and 0.8500, respectively.

A similar improvement was observed for the F1 score. APOEFormer achieved an F1 score of 0.5833, compared with 0.3167 for the MLP, 0.1667 for logistic regression, and 0.0000 for random forest. This indicates that APOEFormer provided a better balance in predicting the APOE4-positive class than the conventional baseline models.

For probability-based discrimination, APOEFormer achieved an AUROC of 0.7250 and an AUPRC of 0.8500. Both values were the highest among all four methods. The AUPRC values for the MLP, random forest, and logistic regression were 0.7917, 0.7750, and 0.7500, respectively, while their AUROC values were 0.6750, 0.7125, and 0.6000, respectively.

The random forest results illustrate the distinction between ranking-based and threshold-based performance. Although random forest achieved a mean AUROC of 0.7125, its mean accuracy was 50.0% and its F1 score was 0.0000 at the fixed classification threshold of 0.5. This suggests that the model could rank some APOE4-positive patients above APOE4-negative patients without assigning probabilities sufficiently high to classify the positive samples correctly at the selected threshold. Because each test set contained only four patients, AUROC and AUPRC should also be interpreted cautiously, as the ranking of a small number of samples can substantially affect these metrics.

Overall, APOEFormer achieved the best performance in terms of accuracy, F1 score, AUROC, and AUPRC among the evaluated methods. These findings indicate that the predictive performance of APOEFormer was not reproduced by simply concatenating the longitudinal patient features and applying standard linear, neural-network, or tree-based classifiers. Nevertheless, the small cohort size and resulting variability across runs motivate further evaluation using larger independent patient cohorts.

### 3.4. Ablation Study of Contrastive Pretraining

To evaluate the contribution of contrastive pretraining to APOEFormer, we performed an ablation experiment in which the same model architecture was trained without the contrastive pretraining stage. All other components of the model, including the patient-level training, validation, and test partitions, were kept unchanged so that the effect of contrastive pretraining could be isolated. Contrastive representation learning has previously been shown to improve the quality of learned representations and downstream performance, particularly when labeled data are limited [15].

The full APOEFormer model with contrastive pretraining was compared with the same architecture trained without pretraining across the same 10 experimental runs. Table 5 summarizes the resulting test performance.

Contrastive pretraining improved performance across all four evaluation metrics. The mean test accuracy increased from 57.5% without pretraining to 75.0% with pretraining, corresponding to an improvement of 17.5 percentage points. The F1 score increased from 0.4033 to 0.5833, while AUROC improved from 0.5250 to 0.7250. AUPRC also increased from 0.7000 without pretraining to 0.8500 with pretraining.

The confidence intervals further reflect the variability associated with the small cohort. Without contrastive pretraining, APOEFormer achieved an accuracy of 0.5750 (95% CI: 0.4053–0.7447), an F1 score of 0.4033 (95% CI: 0.1302–0.6765), an AUROC of 0.5250 (95% CI: 0.3109–0.7391), and an AUPRC of 0.7000 (95% CI: 0.5646–0.8354). In comparison, the model with contrastive pretraining achieved an accuracy of 0.7500 (95% CI: 0.5814–0.9186), an F1 score of 0.5833 (95% CI: 0.2692–0.8975), an AUROC of 0.7250 (95% CI: 0.4795–0.9705), and an AUPRC of 0.8500 (95% CI: 0.7126–0.9874).

These results indicate that contrastive pretraining provides a meaningful empirical performance benefit for APOEFormer in this limited-sample setting. In particular, the consistent improvement in accuracy, F1 score, AUROC, and AUPRC suggests that the representations learned during the contrastive stage are more informative for subsequent APOE4 carrier-status classification than representations learned solely through supervised training. However, because of the small cohort size and overlapping confidence intervals, these findings should be interpreted as evidence of improved empirical performance rather than definitive statistical significance.

### 3.5. Predictive Power of Individual Modalities

To measure the standalone predictive capability of each data type for APOE4 carrier status, we conducted an ablation study to evaluate the predictive performance of APOEFormer using each modality independently. This approach has been widely used in multimodal biomedical learning to disentangle the relative contribution of heterogeneous inputs and to identify dominant sources of predictive signal [11,13].

The results, summarized in Figure 3, reveal clear differences in predictive strength across modalities. Blood metabolites achieve the highest standalone accuracy (62%) among all modalities, indicating that circulating metabolic profiles contain strong information associated with APOE ε4-related biological processes. This finding is consistent with prior studies demonstrating links between APOE4 genotype and lipid metabolism, mitochondrial function, and systemic metabolic alterations [5,25].

Other blood-based modalities, including CBC value and inflammatory markers, also exhibit limited standalone predictive performance (accuracy between 48% and 50%). This observation aligns with evidence that APOE4 carriage is associated with altered immune responses and chronic inflammation, which can be reflected in peripheral blood biomarkers [43]. Together, these results suggest that peripheral biochemical and immune-related signals may contribute to the characterization of phenotypic patterns associated with APOE4 carrier status.

Structural MRI is the second most predictive modality when evaluated in isolation (accuracy = 55%). Among neuroimaging modalities, it is the most informative, outperforming perfusion image (accuracy = 48%) and CBF value (accuracy = 42%). Structural MRI has been extensively shown to capture APOE4-related patterns of brain atrophy, particularly in medial temporal and cortical regions, even in cognitively normal individuals [44]. In contrast, perfusion image and CBF value provide complementary measures of cerebral physiology but exhibit weaker discriminative performance when used alone, suggesting that functional alterations may be more subtle or context-dependent.

Microbiome data demonstrates limited standalone performance (accuracy = 50%), the same as CBC value. While emerging evidence suggests an association between gut microbiota composition, neuroinflammation, and Alzheimer’s disease risk, these relationships may be influenced by numerous confounding factors [45]. The observed performance is therefore consistent with the microbiome serving as a secondary or modulatory signal associated with APOE4 carrier status rather than a strong standalone predictor. Demographic/clinical data—including variables such as age, sex, and body mass index (BMI)—also demonstrates limited standalone predictive capability (50%). These variables may capture indirect population-level associations with APOE4 status but lack strong discriminative power when used in isolation. In contrast, CBF value exhibits the lowest accuracy (42%), suggesting limited standalone discriminative performance.

Finally, although individual modalities provide varying degrees of predictive information, no single modality achieved the 75% classification accuracy obtained through multimodal integration, supporting the use of combined multimodal information for APOE4 carrier-status classification.

### 3.6. SHAP-Based Analysis of Modality Importance in Multimodal Integration

To complement the individual modality analysis, we examined the relative contribution of each modality using the SHAP importance scores obtained from the trained multimodal model.

As summarized in Figure 4, the SHAP results have some similarity with the trends observed in the ablation study. Blood metabolite data exhibits the highest average SHAP importance (0.449), indicating that it contributes most strongly to the model’s predictions. Other blood-based modalities, including general CBC value (0.357) and inflammatory markers (0.339), also receive relatively high SHAP values, reinforcing their central role in APOE4 classification.

Microbiome features also show relatively high SHAP importance (0.346), indicating they are complementary with other features in multimodal data integration.

Neuroimaging modalities—including structural MRI, and perfusion imaging—exhibit moderate SHAP importance, lower than that of blood metabolites, CBC value and microbiome. These findings suggest that imaging features play a supplementary role by refining and contextualizing predictions derived from biochemical signals in multimodal data integration, rather than serving as the dominant source of information. This interpretation is consistent with prior multimodal AD studies, where imaging features enhance prediction when combined with molecular or clinical biomarkers [11,13].

It is worth noting there is some difference in the relative importance of data modalities in the multimodal integration setting (Figure 4) and in the isolation setting (Figure 3). For instance, the relative importance of structural MRI in multimodal data integration is lower than its importance when it is used alone. The difference is because the former consider the interactions between modalities while the latter measure the absolute predictive power of each modality.

Importantly, the results indicate that blood-based modalities—particularly metabolite and inflammatory measurements—constitute the primary source of predictive signal for integrative APOE4 classification, while microbiome features provide complementary context. Structural and functional neuroimaging modalities further enrich the multimodal representation, supporting the rationale for integrating diverse data sources even when individual modalities are only moderately informative.

### 3.7. Identifying Brain Regions Associated with APOE4

Table 6 summarizes the top-ranked structural MRI brain regions with the highest average absolute importance following feature normalization. Out of nearly 170 anatomical regions evaluated in the structural MRI feature set, only the most influential regions are reported for clarity and interpretability. The top-ranked regions span medial cortical areas, frontal cortex, temporal lobe structures, parietal regions, subcortical nuclei, cerebellar white matter, and interhemispheric white matter tracts. The top five brain regions are also highlighted in Figure 5. The diversity of regions represented among these leading features indicates that model predictions rely on distributed anatomical information rather than localized structural effects.

The isthmus of the cingulate gyrus (wm-rh-isthmuscingulate) emerged as the most influential region in the normalized ranking. This region forms part of the medial cortical system and is anatomically positioned at the interface between posterior cingulate and medial temporal areas, supporting integrative cognitive and internally directed processes. Prior neuroimaging work has consistently identified the posterior cingulate and adjacent medial parietal regions as central hubs within large-scale brain networks involved in memory and self-referential cognition [46,47]. The prominence of the isthmus cingulate in the importance analysis therefore reflects APOE4 prediction sensitivity to structural variation within widely connected medial cortical networks.

Several frontal regions, including the pars triangularis, also ranked among the most influential features. The pars triangularis is a subdivision of the inferior frontal gyrus and has been implicated in higher-order cognitive and language-related functions in functional and structural neuroanatomical studies [48]. Its contribution suggests that frontal cortical morphology may provide complementary predictive information.

Additional regions appearing among the top-ranked parcels include the temporal pole, precuneus, cuneus, thalamus, putamen, pallidum, cerebellar white matter, and posterior corpus callosum. These regions are commonly involved in memory-related processing, visuospatial integration, subcortical–cortical communication, and interhemispheric connectivity in the healthy brain [47]. Their collective presence among influential features further supports the interpretation that the model leverages broad anatomical variation across multiple functional systems.

Across the full MRI parcellation, importance values exhibited a gradual decay rather than a sharp cutoff, indicating that no single region dominates the prediction process. This pattern suggests that APOE4 prediction from structural MRI is driven by coordinated information across multiple brain regions. Feature normalization plays a key role in revealing this distributed structure by mitigating biases related to parcel size and variance magnitude.

Overall, the structural MRI-based importance analysis indicates that model predictions are informed by anatomically meaningful but spatially distributed brain regions. The alignment between regions emphasized by the model and well-established large-scale brain networks supports the interpretability of the MRI component of the framework, while remaining agnostic to specific disease mechanisms or regional causality.

### 3.8. Identifying Key Blood Metabolites for APOE4 Prediction

Table 7 summarizes the top-ranked blood metabolites identified through the perturbation-based importance analysis. The most influential features span diverse biochemical classes, including metabolites related to energy metabolism, amino acid turnover, nucleotide synthesis, and neurotransmission, suggesting that model predictions draw on distributed metabolic information rather than a single dominant pathway.

Creatinine exhibited the largest average perturbation effect. As a downstream product of creatine metabolism, circulating creatinine is commonly interpreted as a marker of systemic energy metabolism and renal clearance rather than a direct indicator of neural function. Prior studies have reported associations between altered brain energy metabolism, mitochondrial efficiency, and neurodegenerative processes, including AD, particularly in populations at elevated genetic risk [49]. In this context, the influence of creatinine in the model likely reflects broader metabolic or physiological states correlated with disease risk, rather than a specific mechanistic role.

Inositol also ranked among the most influential metabolites. Inositol participates in phosphatidylinositol signaling pathways that regulate membrane dynamics and intracellular signaling across many tissues. Altered inositol levels have been observed in neuroimaging and biochemical studies of neurodegenerative conditions, often interpreted as reflecting glial activity or changes in cellular signaling environments. However, such alterations are not specific to AD and should be viewed as indicators of general metabolic or cellular state rather than disease-specific markers.

Fructose-6-phosphate, a central intermediate in glycolysis and related biosynthetic pathways, showed a notable contribution to model predictions. Disruptions in glucose metabolism and reduced cerebral glucose utilization are well-documented features of AD at the systems level, including in asymptomatic individuals at genetic risk [49]. The importance of glycolytic intermediates in the model is therefore consistent with prior observations of metabolic vulnerability in neurodegeneration, while remaining nonspecific to brain tissue or pathology.

Additional metabolites with elevated importance—including succinate, malate, glycerol, and erythrose—are involved in mitochondrial respiration and intermediary metabolism, while amino acids such as glycine, threonine, proline, methionine, tyrosine, and tryptophan reflect protein turnover and neurotransmitter precursor availability. Nucleotide-related metabolites such as uridine further reflect cellular maintenance and biosynthetic activity. Broad alterations in mitochondrial metabolism, amino acid homeostasis, and neurotransmitter balance have been reported in AD and related neurodegenerative disorders, although these changes are typically systemic and multifactorial rather than disease-specific [50,51].

### 3.9. Identifying Key CBC Value Measurements for APOE4 Prediction

Table 8 reports the most influential blood-based laboratory variables as quantified by the perturbation-based mean change analysis. The identified features span renal, hepatic, metabolic, hematological, and immune-related processes, underscoring the contribution of systemic physiological states to APOE4 prediction and AD-related risk modeling.

Markers of renal and metabolic function, including estimated glomerular filtration rate (GFR), blood urea nitrogen (BUN), and the anion gap, ranked among the most influential variables. Impaired renal function and metabolic imbalance are increasingly recognized as contributors to cognitive decline, potentially through shared vascular pathology, chronic inflammation, and toxin clearance dysfunction. Epidemiological and clinical studies have reported associations between reduced kidney function and increased risk of dementia and AD, supporting the relevance of these systemic markers in neurodegenerative risk stratification [52].

Liver-associated enzymes, particularly aspartate aminotransferase (AST) and alanine aminotransferase (ALT), also exhibited measurable influence. Although these enzymes are not specific to central nervous system pathology, they serve as indicators of systemic metabolic status and mitochondrial stress. Growing evidence suggests that metabolic dysregulation and mitochondrial dysfunction play important roles in AD pathophysiology, including impaired energy metabolism, oxidative stress, and neuronal vulnerability [50,53].

Several hematological indices—including hemoglobin concentration, platelet count (PLT), red cell distribution width (RDW), and mean platelet volume (MPV)—were likewise influential. Alterations in blood cell morphology and platelet activity have been linked to cerebrovascular dysfunction and systemic inflammation, both of which are implicated in AD progression. In particular, elevated RDW and abnormal platelet activation have been associated with cognitive impairment and neurodegenerative disease in prior studies [54].

Immune-related measures, including eosinophil, neutrophil, and granulocyte counts, further highlight the role of peripheral immune activity. Chronic systemic inflammation is a well-established contributor to AD, influencing microglial activation, amyloid pathology, and neurodegenerative cascades. Importantly, inflammatory processes interact with APOE4-associated risk mechanisms, amplifying susceptibility to neurodegeneration [43,55].

These results demonstrate that APOEFormer captures distributed signals across multiple physiological systems rather than relying on isolated biomarkers. The integration of standard clinical laboratory features with metabolomic and imaging-derived representations aligns with the multifactorial and systemic nature of AD, supporting the use of multimodal learning frameworks for robust modeling of genetic phenotypes.

## 4. Discussion

In this study, we introduce APOEFormer, a multimodal transformer for predicting APOE4 carrier status through the integration of heterogeneous biomedical data, including structural brain MRI, Perfusion image, CBF value, blood-based biomarkers, gut microbiome profiles, and auxiliary demographic/clinical variables. APOEFormer adopts a two-stage learning paradigm in which modality-specific encoders are first pretrained and aligned via contrastive representation learning, followed by multimodal integration using a transformer architecture for subject-level prediction. Transformer-based attention enables flexible modeling of cross-modal dependencies without imposing a fixed ordering over modalities, making it particularly well suited for heterogeneous biomedical inputs where modality order is not inherently defined [18,56].

Across 10 repeated experimental runs using different patient-level data partitions, APOEFormer achieved a mean classification accuracy of 75% for APOE4 carrier prediction on the test data. Importantly, the union of the test sets across runs covered all 23 subjects in the cohort, providing broader held-out subject coverage than evaluation using a single fixed test partition. However, because the repeated runs were generated from overlapping partitions of the same small cohort, they should not be interpreted as statistically independent evaluations. The observed run-to-run variability therefore reflects sensitivity to different patient-level data partitions rather than evidence of independent generalization. Validation in larger, independent cohorts is required to assess the robustness and generalizability of the model.

A central finding of this work is the importance of multimodal biological integration for APOE4 prediction and characterization. Both modality-wise ablation experiments and SHAP-based interpretability analyses consistently identified blood-based modalities—particularly metabolite markers—as the strongest contributors to predictive performance. This observation aligns with prior evidence linking APOE4 to systemic metabolic dysregulation, altered lipid metabolism, immune activation, and chronic inflammation [25,57]. Structural MRI emerged as the second most informative imaging modality when evaluated independently. Rather than acting as the primary driver of predictions, imaging features provided complementary anatomical context that refined predictions derived from biochemical signals. Microbiome and auxiliary clinical variables exhibited also contributed meaningfully to the prediction when used in isolation or within the transformer-based multimodal fusion setting.

Perturbation-based analyses revealed that influential features span energy metabolism, amino acid turnover, mitochondrial intermediates, renal and hepatic function, hematological indices, and immune-related markers, indicating that predictive signals arise from distributed systemic physiology rather than isolated biomarkers.

At the metabolite level, highly ranked features included creatinine, inositol, glycolytic intermediates, tricarboxylic acid cycle metabolites, amino acids, and neurotransmitter-related compounds. These results suggest that global metabolic state—including energy utilization, mitochondrial function, and biosynthetic activity—provides meaningful context for genetic risk modeling. Importantly, the model does not rely on any single metabolite, but instead integrates coordinated signals across multiple metabolic pathways, consistent with the multifactorial and systemic nature of neurodegenerative vulnerability.

Complementary patterns were observed in blood-based laboratory features, particularly within the CBC value modality, where markers of renal function, hepatic metabolism, hematological status, and immune activity contributed to predictive performance. The prominence of measures such as glomerular filtration rate, liver enzymes, red blood cell indices, platelet-related metrics, and inflammatory cell counts further supports the interpretation that peripheral physiological states reflect biologically relevant context for APOE4-associated risk. These findings align with prior evidence that metabolic dysregulation, vascular health, and immune activation interact with genetic susceptibility in AD, while remaining nonspecific to any single disease mechanism.

Despite the encouraging performance and interpretability results, several limitations of the proposed framework should be acknowledged. First, the relatively small cohort size restricts statistical power and increases variance in performance estimates, even when using repeated data splits. Although the repeated evaluation protocol mitigates overfitting to individual partitions, performance metrics should be interpreted cautiously and validated in larger, independent cohorts.

Future work should also evaluate a more restrictive imaging pipeline in which image representations are extracted using a completely frozen pretrained vision encoder and subsequently treated as fixed inputs to a lightweight multimodal fusion model. Approaches based on concatenation or cross-attention may further reduce trainable model complexity and the risk of overfitting in small-cohort settings.

Second, the perturbation-based interpretability analyses quantify feature influence within the trained model but do not establish causal relationships between specific biological variables and APOE4 carrier status. Highly ranked metabolites and blood laboratory measures should therefore be interpreted as correlates rather than mechanistic drivers, and further experimental or longitudinal studies are required to disentangle causality.

Third, the feature-importance and brain-region rankings should be interpreted cautiously because they were derived from a relatively large number of candidate features using a small cohort of 23 patients. The analysis included a large set of MRI regions, metabolites, and laboratory features, and no formal correction for multiple comparisons was applied to the resulting importance rankings. Consequently, some highly ranked features may reflect variability associated with the limited sample size rather than stable biological effects. The reported rankings should therefore be considered exploratory and hypothesis-generating rather than definitive. Future studies with larger cohorts should evaluate the stability of these rankings across independent datasets and quantify uncertainty using approaches such as bootstrap resampling or repeated cross-validation.

## 5. Conclusions

In this study, we presented APOEFormer, a two-stage multimodal learning framework that combines contrastive representation learning with transformer-based integration to characterize phenotypic patterns associated with APOE4 carrier status. By integrating structural MRI, perfusion imaging, blood-based biomarkers, metabolomic profiles, microbiome data, and demographic/clinical variables, APOEFormer achieved a mean classification accuracy of 75.0% across 10 repeated patient-level data partitions. Comparisons with the implemented conventional machine-learning baselines showed that APOEFormer achieved higher mean accuracy, F1 score, AUROC, and AUPRC than the evaluated baseline models. The contrastive-pretraining ablation further demonstrated higher empirical performance with pretraining than without pretraining.

Beyond prediction, the interpretability analyses identified contributions from multiple biological domains, including neuroimaging, metabolic, hematological, and other systemic physiological features. These findings support the use of multimodal learning as a tool for investigating phenotypic patterns associated with genetic risk. Importantly, APOEFormer is not intended to replace direct genetic testing; rather, its value lies in characterizing how APOE4-associated genetic risk may be reflected across heterogeneous observable phenotypes.

Nevertheless, the findings should be considered preliminary because of the small cohort size, variability across repeated data partitions, and lack of external validation. In addition, the identified feature and brain-region importance rankings are exploratory and should not be interpreted as causal biological associations. Future work should validate the framework in larger independent cohorts, assess the stability of the identified multimodal signatures, and investigate whether similar representation-learning strategies can support the characterization of other disease-associated genetic and phenotypic patterns.

## Figures and Tables

**Figure 1:**
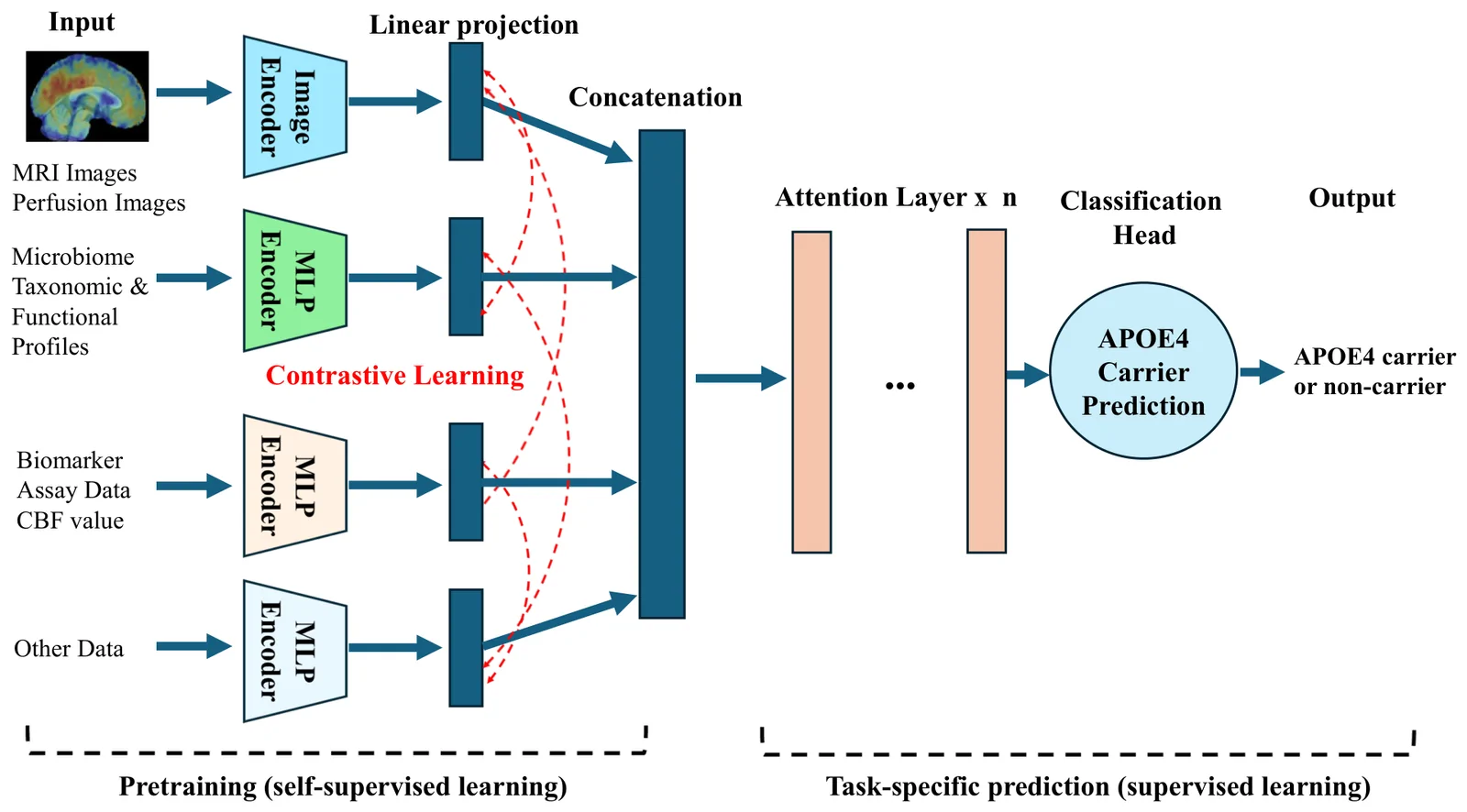
Overview of APOEFormer—a two-stage multimodal AI framework for Alzheimer’s disease analysis. Modality-specific encoders are first aligned using contrastive learning (indicated by the red dotted arrows) to produce shared latent representations from imaging and non-imaging data. Different colors are used to distinguish the input modalities and their corresponding modality-specific encoders for visual clarity. The resulting representations are then integrated by a transformer-based fusion module (indicated by the ellipsis) to predict the presence of an APOE4 allele (APOE4 carrier or non-carrier).

**Figure 2:**
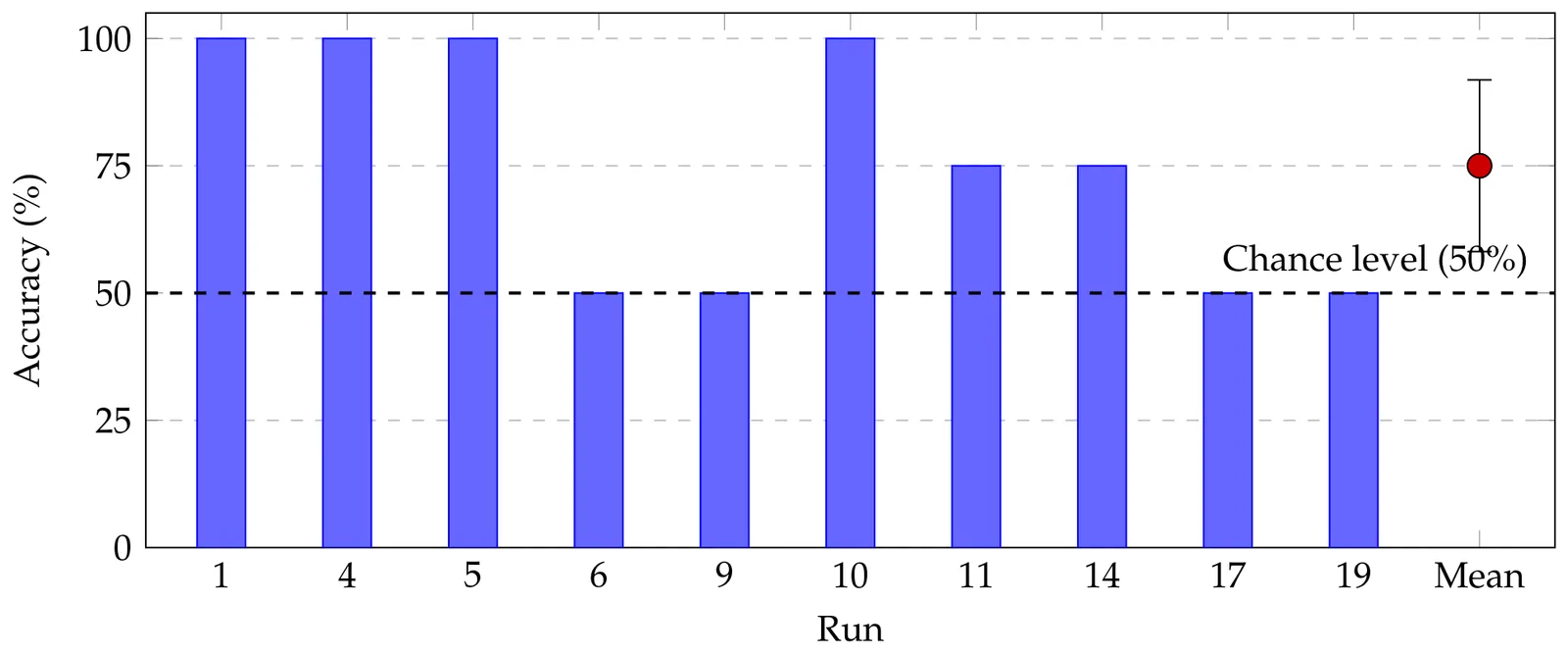
APOE4 classification accuracy across 10 repeated experimental runs. The dashed horizontal line indicates the 50% chance-level accuracy for binary classification. The point labeled “Mean” represents the mean accuracy across the 10 runs (75.0%), with the error bar indicating the approximate 95% confidence interval for the mean (58.14–91.86%) calculated using the Student’s *t* distribution.

**Figure 3:**
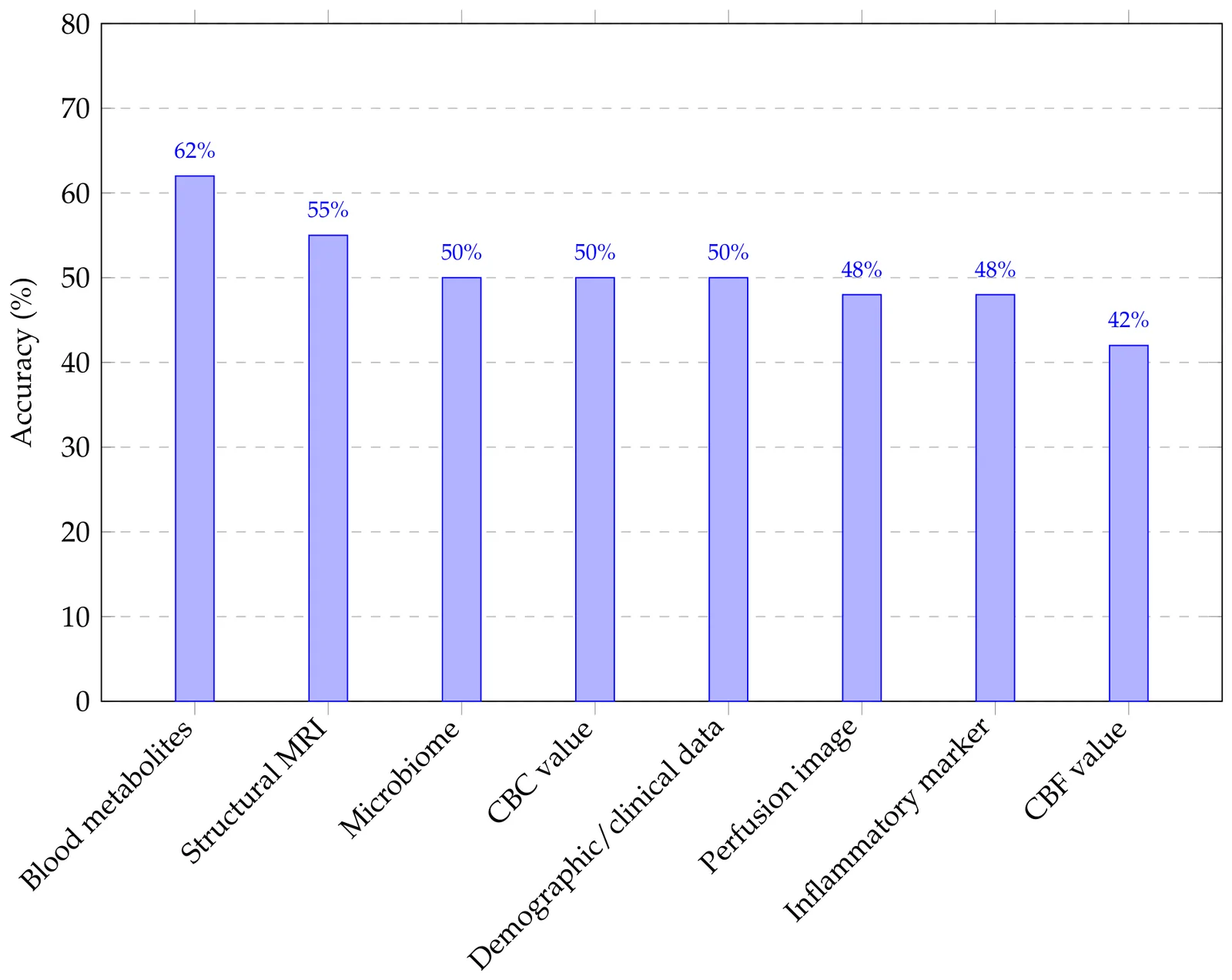
Accuracy obtained when each modality is used individually for model training.

**Figure 4:**
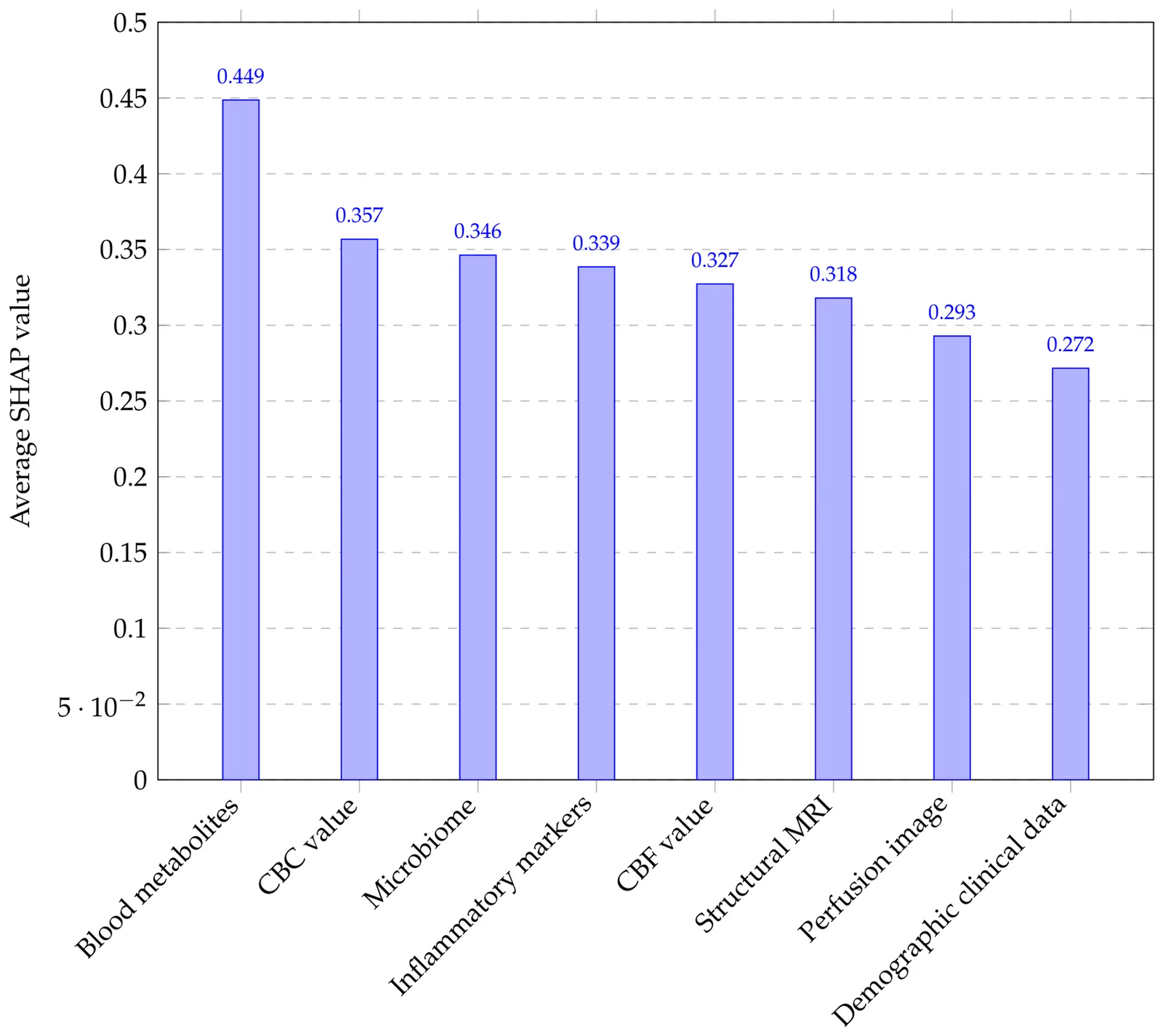
Average absolute SHAP values across input modalities, indicating the relative contribution of each modality to model predictions when they are integrated together.

**Figure 5:**
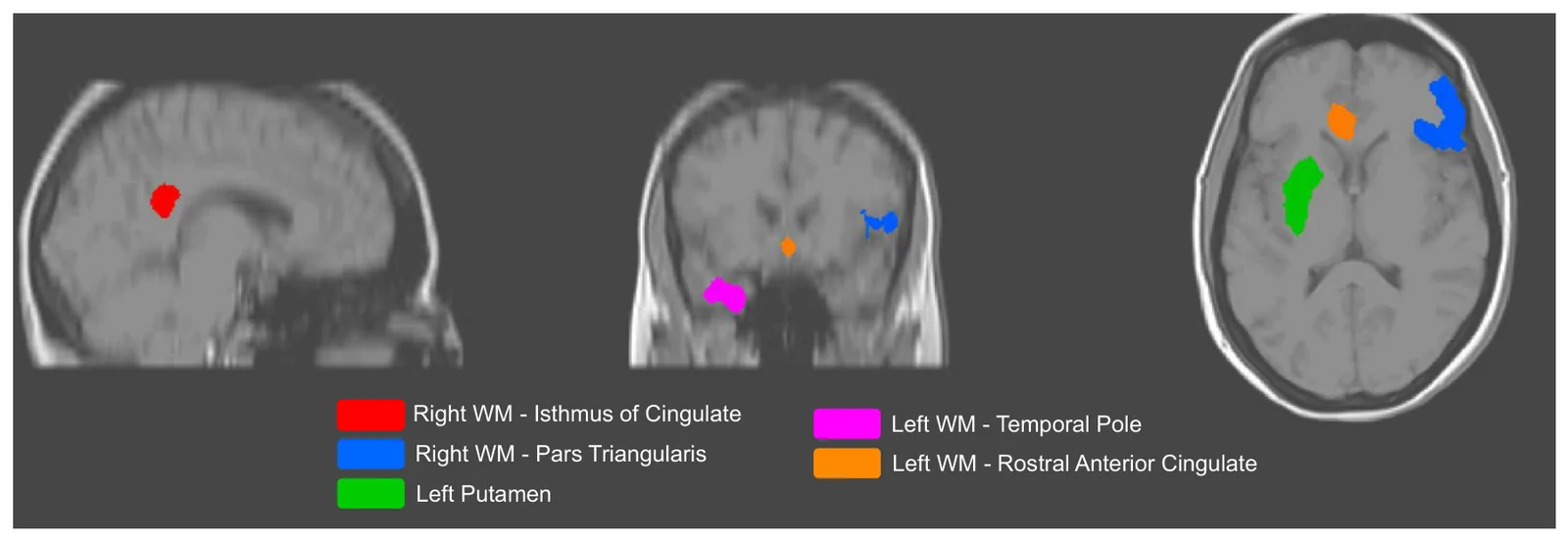
Visualization of the top five structural MRI regions ranked by average absolute mean change (Table 6). Highlighted regions correspond to: Right white matter—Isthmus of the cingulate; Right white matter—Pars triangularis; Left putamen; Left white matter—Temporal pole; and Left white matter—Rostral anterior cingulate. Regions are overlaid on a representative participant’s T1-weighted MRI for spatial interpretability.

**Table 1:** Main architectural and training hyperparameters used for APOEFormer.

Hyperparameter	Value
Batch size	128
Embedding dimension	128
Number of transformer layers	3
Number of attention heads	2
Dropout rate	0.3
Contrastive temperature τ	0.1
Focal-loss $\alpha_{\mathrm{pos}}$	0.9
Focal-loss $\alpha_{\mathrm{neg}}$	0.1
Focal-loss γ	7.0
Contrastive learning rate	$5\times 10^{-3}$
Projection-head learning rate	$5\times 10^{-3}$
MLP learning rate	$5\times 10^{-3}$
Maximum fine-tuning epochs	1000
Fine-tuning early-stopping patience	500
BCE auxiliary-loss weight $\lambda_{\mathrm{CE}}$	0.05
Classification threshold	0.5
Ensemble size	1
Number of experimental runs	10

**Table 2:** Split-level variability in APOE4 classification performance across the 10 repeated experimental runs. Values are reported as mean, standard deviation (SD), range, and approximate 95% confidence interval (CI) for the mean.

Metric	Mean	SD	Range	95% CI
Accuracy	0.750	0.236	0.500–1.000	0.581–0.919
F1 score	0.583	0.439	0.000–1.000	0.269–0.897
AUROC	0.725	0.343	0.000–1.000	0.480–0.970
AUPRC	0.850	0.192	0.417–1.000	0.713–0.987
Sensitivity	0.550	0.438	0.000–1.000	0.237–0.863
Specificity	0.950	0.158	0.500–1.000	0.837–1.063
Balanced accuracy	0.750	0.236	0.500–1.000	0.581–0.919

**Table 3:** Subject-level statistical evaluation of APOEFormer after aggregating repeated held-out predictions across the 10 experimental runs.

Metric	Value
Number of subjects	23
Correctly classified subjects	18
Subject-level accuracy	78.3%
95% exact binomial CI	56.3–92.5%
Chance-level reference	50.0%
Exact binomial test *p*-value	0.0053
Majority-class baseline accuracy	60.9%
McNemar discordant pairs (b,c)	(5,1)
Exact McNemar test *p*-value	0.2188

**Table 4:** Comparison of APOEFormer with conventional machine-learning baselines across 10 patient-level data splits. Values represent mean test performance across the 10 runs. The best result for each metric is shown in bold.

Model	Accuracy	F1-Score	AUROC	AUPRC
Logistic Regression	0.5000	0.1667	0.6000	0.7500
MLP	0.5500	0.3167	0.6750	0.7917
Random Forest	0.5000	0.0000	0.7125	0.7750
**APOEFormer**	**0.7500**	**0.5833**	**0.7250**	**0.8500**

**Table 5:** Ablation study evaluating the contribution of contrastive pretraining to APOEFormer across 10 patient-level data splits. Values represent mean test performance across the 10 runs.

Model	Accuracy	F1-Score	AUROC	AUPRC
Without Pretraining	0.5750	0.4033	0.5250	0.7000
With Pretraining	0.7500	0.5833	0.7250	0.8500

**Table 6:** Top brain regions ranked by average absolute importance.

Region	Average Absolute Mean Change
Right Hemisphere White Matter—Isthmus of the Cingulate Gyrus	0.000769
Right Hemisphere White Matter—Pars Triangularis	0.000569
Left Putamen	0.000550
Left Hemisphere White Matter—Temporal Pole	0.000538
Left Hemisphere White Matter—Rostral Anterior Cingulate Cortex	0.000494
Left Hemisphere White Matter—Cuneus	0.000489
Left Hemisphere White Matter—Pars Triangularis	0.000469
Posterior Corpus Callosum	0.000440
Left Hemisphere White Matter—Isthmus of the Cingulate Gyrus	0.000438
Right Hemisphere White Matter—Cuneus	0.000432
Right Cerebellar White Matter	0.000414
Right Hemisphere Cortex—Isthmus of the Cingulate Gyrus	0.000409
Right Hemisphere White Matter—Precuneus	0.000370
Left Pallidum	0.000369
Left Hemisphere Cortex—Temporal Pole	0.000340
Right Thalamus	0.000339
Right Hemisphere White Matter—Rostral Anterior Cingulate Cortex	0.000319

**Table 7:** Top metabolites ranked by average absolute importance. Among the 27 quantified metabolites included in the analysis, only the highest-ranked features are shown.

Metabolite	Mean Change
Creatinine	0.008487
Inositol	0.007238
Fructose-6-phosphate	0.005095
Threonine	0.004370
Succinate	0.003907
Uridine	0.003879
Proline	0.003756
Pterin	0.003738
Erythrose	0.003425
Glycine	0.003168
Gamma-aminobutyrate	0.003141
Tyrosine	0.003030
Glycerol	0.002733
Tryptophan	0.002499
Methionine	0.002415
Malate	0.002377

**Table 8:** Top blood laboratory features ranked by perturbation-based mean change. Among the 46 laboratory variables included in the analysis, only the highest-ranked features are shown.

Feature	Mean Change
AST (units/L)	0.000886
GFR	0.000502
Anion gap (mmol/L)	0.000158
Potassium (mmol/L)	0.000082
Abs Eosinophils $\times 10^{9}$/L	0.000055
% Eosinophils	0.000030
MPV	0.000019
ALT (units/L)	0.000019
Sirolimus Lvl (ng/mL)	0.000019
HGB (g/dL)	0.000017
% Neutrophils	0.000015
PLT $\times 10^{9}$/L	0.000014
Triglycerides (mg/dL)	0.000012
Abs Granulocytes $\times 10^{9}$/L	0.000012
RDW CV (%)	0.000011
BUN (mg/dL)	0.000011
Hemoglobin A1c (%)	0.000010

## Data Availability

The source code and documentation associated with APOEFormer (version 1.0) are available at https://github.com/jianlin-cheng/APOEFormer (accessed on 21 September 2026). The underlying patient-level MRI, metabolomic, and microbiome data are not publicly accessible and are not distributed through the GitHub repository.
